# Promoting Motor Function by Exercising the Brain

**DOI:** 10.3390/brainsci3010101

**Published:** 2013-01-25

**Authors:** Stephane Perrey

**Affiliations:** Movement to Health (M2H), EuroMov, Montpellier-1 University, 700 avenue du pic saint loup, 34090 Montpellier, France; E-Mail: stephane.perrey@univ-montp1.fr; Tel.: +33-411-759-066; Fax: +33-411-759-050

**Keywords:** cerebral oxygenation, endurance exercise, hemodynamics, neuroplasticity, cerebral blood flow

## Abstract

Exercise represents a behavioral intervention that enhances brain health and motor function. The increase in cerebral blood volume in response to physical activity may be responsible for improving brain function. Among the various neuroimaging techniques used to monitor brain hemodynamic response during exercise, functional near-infrared spectroscopy could facilitate the measurement of task-related cortical responses noninvasively and is relatively robust with regard to the subjects’ motion. Although the components of optimal exercise interventions have not been determined, evidence from animal and human studies suggests that aerobic exercise with sufficiently high intensity has neuroprotective properties and promotes motor function. This review provides an insight into the effect of physical activity (based on endurance and resistance exercises) on brain function for producing movement. Since most progress in the study of brain function has come from patients with neurological disorders (e.g., stroke and Parkinson’s patients), this review presents some findings emphasizing training paradigms for restoring motor function.

## 1. Introduction

Maintaining brain health and plasticity throughout life is an important public health goal, and the correlation between exercise and behavioral stimulation in supporting this achievement is clear. Indeed, research on human factors and physical activity suggests that exercise can maintain or even improve brain plasticity [1]. Plasticity is here defined as the normal ongoing state of the nervous system throughout one’s life span, while behavior is the manifestation of the coordinated workings of the entire nervous system. Whereas exercise is the intentional engagement in physical activity in order to enhance fitness level, physical activity encompasses all forms of movement produced by skeletal muscles [2]. Exercise, as a “stressor,” appears to be a powerful determinant of brain (neuro)physiology [1] and can be viewed as a widely practiced behavior that activates molecular and cellular cascades which support and maintain brain plasticity. These changes are mediated by upregulation of several growth factors, including vascular endothelial grow factor, and brain-derived neurotrophic factor (BDNF) [3]. BDNF is a key protein in regulating maintenance, growth, and even survival of neurons [4], and has been shown to be an important activity-dependent modulator of synaptic transmission and, in turn, of synaptic plasticity [5]. Furthermore, BDNF appears to mediate exercise-induced neurogenesis, *i.e.*, the process by which new neurons proliferate and develop [6].

Exercise as an acute stressor is known to result in immediate elevations in heart rate, oxygen uptake (VO_2_), respiration, blood flow and many metabolic substrates. However, exercise can also promote physiological changes in the brain. Past transcranial Doppler studies reported that aerobic exercise might be beneficial for the maintenance of cerebral blood flow (CBF) (e.g., [7]). The close relationship between CBF and brain function was first observed in the late nineteenth century by the Italian physiologist, Mosso [8], then by Roy and Sherrington [9]. The authors attributed task-induced vasodilatation to an increased demand for cerebral metabolism in response to neuronal activity. During physical activity, CBF increases to supply adequate oxygen to the brain [10], and the regulation of CBF appears crucial for the maintenance of cardiovascular homeostasis. When experienced multiple times throughout the day, week, or over a month and on to many years, exercise responses (chronic) have also the potential to influence the brain and motor function. Neuroplasticity refers to the ability of the brain and central nervous system (CNS) to adapt to environmental changes by modifying neural connectivity and function, such as exercise training does. To date, the development of human neuroplasticity via exercise is still not well understood. Autonomic cardiovascular arousal, which is obviously influenced by exercise, has profound effects on the CNS. In a review, Dustman *et al.* [11] noted that the findings from animal studies “*strongly suggest there is a positive relationship between physical exercise and CNS health*,” which occurs because of improved neurotransmitter functioning, increased vascularization, and increased cell hypertrophy and complexity. Moreover, CNS adaptations contribute to enhanced motor function (gains in muscular strength). Because noninvasive methods of probing the connections between the brain and muscles, such as transcranial magnetic stimulation (TMS), have become widely available, determining the exact form of the CNS changes has become the subject of considerable attention over the last few years. TMS is a noninvasive technique that allows stimulation of small brain areas. The study of brain function in the last century through work involving the stimulation of the cortex of animal brains using electrical currents led to the mapping of motor function in animals and, later, in humans. In addition, brain neuroimaging techniques have allowed the study of healthy human subjects in the last two decades. With the development of the neuroimaging techniques like computerized tomography and magnetic resonance imaging (MRI), it is now possible to be more specific as to the location of brain regions involved in motor functions. In addition, the assessment of physiological changes associated with brain activity has become possible by optical methods, such as near-infrared spectroscopy (NIRS). During the last decade, researchers have used NIRS extensively to evaluate cerebral oxygenation and blood volume during various tasks involving cognitive and motor stimulation [12,13]. The primary advantage of NIRS over other techniques such as functional MRI (fMRI) in evaluating cerebral function is that it provides information on oxyhemoglobin (HbO_2_), deoxyhemoglobin (HHb) and total hemoglobin (Hbtot, reflecting local blood volume) with a high temporal sampling density than fMRI, that is, >10 Hz *versus* <1 Hz. Because of the cost effectiveness, portability, and ease of use of NIRS, it is an attractive noninvasive technique for evaluating cerebral function in a variety of experimental conditions and settings, as those encountered during exercise [14,15].

As stated earlier, physical activity and, in particular, acute exercise and training seem to be the key interventions to trigger the processes through which neurotrophins mediate energy metabolism and, in turn, neural plasticity. The primary objective of exercise training is to stress various bodily systems to bring about positive adaptation in order to enhance motor performance. The application of some training principles (e.g., overload, specificity) involves the manipulation of various program design variables, including choice of exercise, training intensity (load and repetition), rest periods between exercises and training frequency and volume. While the aforementioned training principles are employed for both endurance and strength training regimes, the physiological adaptations for both are notably different due to differences in the application of program design variables [16]. Basically, endurance-training programs such as those used for running or cycling typically involve the performance of high-repetition, low-resistance exercise continuously over long periods of time (e.g., 1–3 h). In contrast, strength training typically involves the performance of high-resistance, low-repetition exercises to modulate increases in muscle strength, hypertrophy and motor performance [16,17]. To better determine the most efficacious exercise plan for promoting good motor health in the brain, researchers and clinicians need to attend to the multifaceted nature of the exercise prescription. Clarifying the use of some exercise (endurance, resistance, forced) and related modalities thus appears to be necessary. An important question is whether certain targeted exercises have the potential for improving brain function. The multiple methodological approaches (TMS, NIRS) allow us to examine how aerobic and/or resistance exercise may cause functional changes to the brain and then examine how these changes produce a robust effect on behavior performance. Animal models are necessary to discover the specific chemical and neural changes that occur, and neuroscience techniques (e.g., EEG, fMRI, fNIRS) document functional changes at the systems level in humans.

Exercise is nowadays well recognized as an interventional modality for preventing or even improving brain function (and structural) decline with physical inactivity whether it is associated or not with neurodegenerative diseased state. A sedentary lifestyle with low aerobic fitness is associated with both cardiovascular and cerebrovascular diseases [18]. In contrast, the “exercising” brain is associated with marked structural and functional modifications in the cardiovascular and cerebrovascular systems, which are, in turn, linked to neurophysiological and neuropsychological changes. For the latter, most research supported or at least partially demonstrated a positive effect of submaximal exercise on cognitive performance. One important moderator that might have influenced the cognitive results is exercise intensity. When using a moderate intensity exercise protocol (40% to 60% of the participant’s maximal VO_2_, maximal aerobic output) with a duration from 20 to 60 min, most research supported either a positive effect [19,20] or a slightly positive trend [21,22] for the relationship between exercise and cognition. In addition, most studies testing the effects of incremental whole-body exercise on cognition supported either a linear facilitation [23] or an inverted-U facilitation effect [24,25]. The relationship between physical activity and brain functions has been widely investigated. Many exercise studies primarily focus on brain structural and functional changes relative to cognitive improvements, with relatively few studies focusing on the motor performance. Thus, this review will not focus on the impact of exercise on cognitive function, due to large spectra of reviews on this topic. Rather, this review will concentrate on how motor function is promoted by “exercising” the brain. Particularly, how exercise modalities could impact motor movement and brain function is the main question addressed in this review. Therefore, the purpose of the current review is to provide an insight into the effect of physical activity (based on endurance and resistance exercises) on brain function for producing movement. Since most progress in the study of brain function has come from patients with neurological disorders (e.g., stroke and Parkinson’s patients), this review will present some findings that highlight training paradigms for restoring motor function.

## 2. Neuro-Physiological Processes Associated with Brain Activity When Moving

### 2.1. Organization of the Motor System for Voluntary Movements

**Figure 1:**
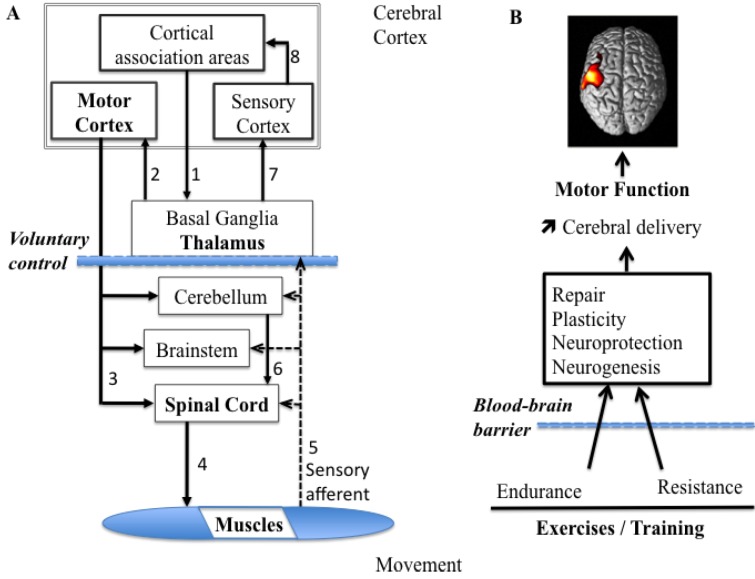
Diagram summarizing the control processes for voluntary movement and the influence of physical exercises on brain plasticity. (**A**) Follow the arrows and numbers from the design to the execution and then the return of feedback. Note that the cortical association areas play the key role in the design and planning of voluntary movements and the thalamus sends impulses to the motor cortex, seen as the final common pathway. Alpha motoneurons cause muscle contraction and relevant feedback information is sent towards the central nervous system (CNS). (**B**) Schematic depicting the main proposed effect of resistance and endurance exercises on CNS function. Both types of exercise lead to improvements in motor function and an increase in the cortical activation due to proper cerebral blood flow and glucose delivery to neurons. Hemodynamic changes (μM)

The ability to produce and modulate force output is an essential aspect of motor control, as tasks performed require the ability to control our force output to accurately suit the tasks at hand. As force production and modulation play such an important role, it is not surprising that there is extensive literature focused on how force is encoded in the healthy brain. When producing a movement, the force output of a skeletal muscle can be changed over a large range [26]. The variety of force profiles and movements depends on both peripheral factors, such as anatomy and fiber type composition of muscles, and central factors, such as the central motor commands. The ability to accurately produce the assorted ranges in force required to control different movements that vary in magnitude, speed and precision is largely accomplished through modifying motor unit patterns [27]. The muscle acts in response to neural commands to produce the required range of motor outputs. Both motor unit recruitment and firing rate modulation are used in combination to produce variations in muscle force [26], and the type of muscle being used, as well as the level of force required, subsequently determine the relative contribution of each. In addition to the motor unit features, the production of voluntary muscle force is the consequence of a number of processes that start in the brain. After a command from the supra-cortical structures, the descending drive from the motor cortical structures activates the spinal motoneurons, which in turn activate muscle fibers (motoneurons) to produce muscle force [28]. Many projections from the cerebral cortex terminate in the brain stem, and others continue through the brain stem and into the spinal cord, where they are called the corticospinal tract (Figure 1A). These projections have several functions, including voluntary control, relay to the cerebellum, activation of other descending pathways, and modulation of sensory processing.

The corticospinal tract represents the highest order of motor function in humans and can be explored by the motor-evoked potential response induced by TMS. In addition to the findings of strength training research (for review see Carroll *et al.* [29]), neuroplasticity occurring in M1 is dependent upon the nature and complexity of the task being performed [30,31,32]. Recent TMS studies have shown changes in corticospinal synaptic activity and/or improved synaptic efficacy of specific intracortical pathways within the M1 following a period of motor skill training [31]. It is noteworthy that the more demanding tasks result in greater activation and higher facilitation than less demanding tasks [31,33].

### 2.2. Optical Imaging of the Brain Function

**Figure 2:**
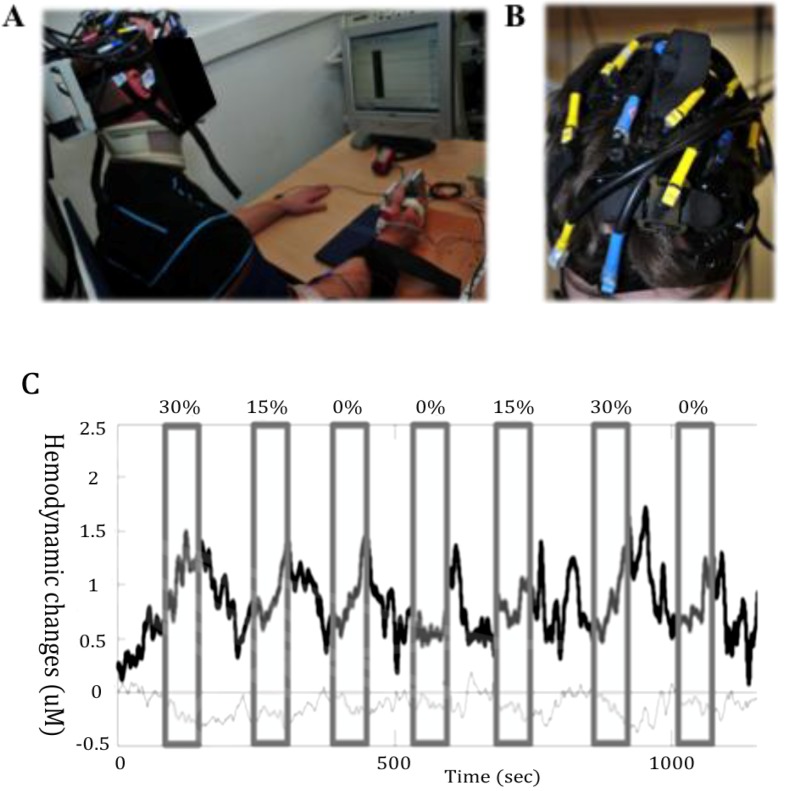
Measurement of cortical activity with near-infrared spectroscopy (NIRS). (**A**) Example of a NIRS setting where the motor response consisted in thumb abduction. (**B**) Illustration of the head coverage setting with a multichannel NIRS instrument during upper body motor tasks. (**C**) A sample of functional NIRS measurement with a 6 Hz sampling rate in a healthy male subject performing in a randomized order handgrip motor tasks during 60 s at 0%, 15% and 30% of his maximal voluntary force. The upper trace (thick) depicts HbO_2_, and the lower trace (thin) HHb. The grey areas depict the duration of motor stimulation. The HbO_2_ increases and the HHb decreases mostly during the stimulation. This corresponds to typical functional cortical activation.

While single cell recordings in non-human primates have provided invaluable insights into how movement is coded in the motor cortex, the recent advances of neuroimaging have helped to further our understanding of the coding of movement in the human brain by examining the dynamic interaction of multiple brain regions. Over the years, a number of different neuroimaging modalities have been developed which have the ability to measure the reorganization of brain function. The human brain is interconnected structurally by a dense network of cortico-cortical axonal pathways, and functionally through synchronized or coherent neural activity [34]. Mapping the brain is crucial to revealing changes that occur in response to neurological disorders. In the last decade, combined TMS-neuroimaging studies have greatly stimulated research in understanding neurophysiological and neuroplastic effects induced by noninvasive brain stimulation [35]. By tracking neural activity in real-time, directly or indirectly, neuroimaging methods help to provide a complex spatiotemporal description of plastic reorganization in humans, which occurs, for instance, following injuries to the nervous system or after physical training. To date, several techniques to examine functional brain activity are available. Historically, electroencepholography (EEG) was the first technology, discovered by the neurologist Hans Berger in the 1920s, followed by other technologies, including positron emission tomography (PET), single photon emission computed tomography (SPECT), magnetencephalography (MEG), and functional magnetic resonance imaging (fMRI). A less known technology for monitoring brain function, NIRS, uses the difference of absorption spectra of oxyhemoglobin and reduced hemoglobin, as well as oxygenated cytochrome oxidase, in the near-infrared. fNIRS relies on the placement of near-infrared light sources and detectors on the scalp (Figure 2). Near-infrared light, particularly that with a wavelength between 650 and 900 nm, can migrate through biological tissues, including skin and skull bone, and can be absorbed by biological chromophores such as hemoglobin when used as a functional brain-imaging tool. The continuous-wave NIRS system measures the transmitted intensity and calculates the relative changes in the hemoglobin concentration according to the modified Beer–Lambert law for highly scattering media.

As mentioned previously, NIRS is more suitable to measure brain activity during motor tasks, making it particularly applicable to neurorehabilitation studies of motor performance [36]. Like fMRI, brain NIRS infers neural activation from the neurally coupled hemodynamic response occurring over 4–6 s. There is a substantial body of literature comparing fNIRS and fMRI (e.g., [37] for a review). A synthesis of the results of these studies indicates that though the signal-to-noise ratio of NIRS measurements is lower, there is nonetheless a strong temporal correlation between signals measured with fNIRS and fMRI in most subjects for various stimulation settings (*i.e.*, motor, visual, cognitive). However, the lack of temporal correlation between the optical and fMRI signals in some subjects may occur, partly due to the contamination of the optical signal by hemodynamic fluctuations in the superficial tissue layers. Recently, Kirilina *et al.* [38] showed that task-evoked hemodynamic changes of veins draining the scalp can induce artifacts in the tissue concentrations of extracranial [HbO_2_], but not [HHb].

### 2.3. Cerebrovascular Changes during Brain Activation

Physiological events associated with brain activity can be subdivided into intracellular events, events occurring at the cell membranes and those that are mediated by neurovascular coupling and occur within the vascular space. In addition to the events taking place intracellularly, local brain activity induces local arteriolar vasodilation [39]. The brain has primarily two primary needs to maintain homeostasis, *i.e.*, oxygen and glucose. Cerebral blood flow has an important role in providing blood supply to the brain. In contrast to other organs, CBF is independent from the changes in cerebral perfusion pressure or arterial blood pressure due to dynamic cerebral autoregulation [40,41]. Dynamic autoregulation not only guarantees adequate oxygen supply, but also forces dilation of the cerebral arterioles [42]. Although small arteries and arterioles probably contain less than 5% of the blood volume in the brain parenchyma, they control most of the resistance and therefore blood flow at a local level [39]. As a consequence of local vasodilation, the local cerebral blood volume (CBV), as well as CBF, increase. This relationship between neuronal activity and vascular response is termed “neurovascular coupling”. This increase in CBF, however, exceeds the increase in oxygen consumption and thus leads to an increase in intravascular hemoglobin oxygenation during brain activity. Therefore, when the NIRS measuring optode is located over an area in which CBF increases during brain activity, a localized increase in the concentration of HbO_2_ and a decrease in deoxygenated hemoglobin HHb is usually seen. Several NIRS studies conducted in the past ten years have demonstrated that activation-induced changes in brain activity can be assessed noninvasively during the performance of motor activity [14,43]. With respect to studies in which NIRS has been employed to evaluate simple and complex motor behaviors in adults, a systematic review has been proposed by Leff *et al.* [44]. Also, the potential of NIRS for exercise applications has been underlined recently [15].

## 3. Brain’s Activation Patterns during Exercise and Training

If one is interested in determining which regions of the brain are being activated/oxygenated during an isolated exercise task, fMRI using the blood-oxygen-level dependent (BOLD) response could be used. For exercise studies involving dynamic movements, the obvious issue to overcome is movement, as most movement causes disruption in the scanning process. Like fMRI, functional NIRS is a hemodynamics-based technique for the assessment of functional activity in the human cortex and has the advantages of being relatively resistant to motion artifacts, allowing easy measurement in less restricted and noisy conditions [14]. The demands placed on the body’s cardiovascular system while exercising (e.g., whole-body exercises, such as walking, running or cycling) induces many physiological changes in skeletal muscle and the brain. This is particularly true when the subject has to sustain a neuromuscular strain at a given load level, or when exercise intensity changes [43]. To date, determination of the regions of activation in the brain during various motor tasks and with exercise training interventions in patients is nascent (e.g., [45]). There is an important need for this kind of research if one desires to gain knowledge of changes in CBV, CBF, and brain activation, due to an exercise stimulus from either an acute exercise bout or in response to a chronic adaptation. Acute and chronic endurance (aerobic) exercises differ in the physiological changes they induce and are discussed in the following. It is noteworthy that resistance exercise involves the voluntary activation of specific skeletal muscles against some form of external resistance, provided by body mass, free weights, or a variety of exercise equipment including machines, springs, elastic bands, or manual resistance. According to the definition of health-related physical fitness, which includes five criteria (cardiovascular ability, muscular strength, muscular endurance, flexibility, and body composition), resistance exercise is a form of exercise that benefits at least three components of health-related physical fitness [46]. It could be likely that resistance exercise might also impact brain function in a fashion similar to that observed with aerobic exercise (Figure 1B). This is because resistance exercise impacts many of the same potential mechanisms that are affected by aerobic exercise and is also a potent stimulus for the release of a variety of neuroendocrine and growth factors from skeletal muscle and other tissues. While resistance exercise increases the torque- and power-generating capacity of the muscle by design, a recent study of Liu-Ambrose *et al.* [47] provided strong evidence with fMRI that resistance training also had a positive effect on functional plasticity in the cortex. Cassilhas *et al.* [48] demonstrated that six months of either moderate- or high-intensity resistance training significantly improved cognitive performance among senior men. Thus, resistance training may promote both cognitive performance and functional plasticity among seniors via mechanisms involving insulin-like growth factor and homocysteine [48]. Further, participation in progressive resistance training enhances BDNF release, similar to the effects of endurance training, and/or that the acute BDNF response to resistance exercise is load dependent [49]. 

### 3.1. Acute Changes Following Exercise

#### 3.1.1. Endurance Exercise

Aerobic dynamic exercise is of interest because the respective physical demands are determined by the capacity of the cardiovascular and respiratory systems, in relation to muscle and cerebral metabolism. Endurance exercise has been prescribed, but the exact intensity of physical activity is still unclear, chiefly the result of methodological issues for monitoring CBV, which has been proposed to be a major determinant of brain function. Previous research on the brain during exercise has focused mainly on cerebral hemodynamics during graded (or incremental) tests. Cardiopulmonary responses to incremental exercise are controlled through a complex combination of feedback and feed-forward mechanisms, including metabolic changes within the active muscle mass and cerebral tissue [42]. Muscle contractions reflexively evoke cardiorespiratory increases via an elevation in the discharge frequency of metabosensitive group III/IV muscle afferents, projecting to the cardiorespiratory control centers in the CNS [50]. This so-called “exercise pressor reflex” (the feedback component) plays, next to central command (the feed-forward component) [51], a key role in the neural control mechanisms determining the proper cardiorespiratory response to exercise. While exercise is known to modify the cardiovascular system, it is not clear how exercise changes CBF. Earlier studies demonstrated that CBF was not affected by various conditions due to cerebral autoregulation. Madsen *et al.* [52] found that no significant change in blood flow during physical activity ensued even though significant changes in cardiac output and blood pressure were evidenced. However, more recent studies indicated that cerebral artery flow velocity and CBF increase during exercise [53,54]. According to a review by Ogoh and Ainslie [42], the varying results between earlier studies and recent studies may be due to different methods to assess CBF. While recent studies used transcranial Doppler ultrasound (TCD), earlier studies used the Kety–Schmidt method (based on application of the Fick principle to the uptake of a tracer—e.g., nitrous oxide—by the brain), may have underestimated the response of cerebral response to exercise. Any increase in CBF during exercise is attributed to increases in cerebral neuronal activity and metabolism [53]. Results from animal and human studies indicate that regional and global cerebral blood flow increases 20%–30% on the transition from rest to moderate exercise, with no further rise when exercise intensity increases to elicit maximal O_2_ uptake [55]. However, the continuous increased blood flow observed in most recent studies during and after exercise to point of exhaustion, together with the increased arterial-internal jugular venous concentration difference for O_2_, glucose and lactate [56] suggest that the metabolic activity of the brain as a whole is enhanced during intense exercise.

Neuronal activation is coupled with increases in regional CBF, which is thought to be accompanied by an increase in CBV via volumetric expansion in vessels already perfused (vasodilatation) or by increasing the portion of vessels actually perfused (recruitment) [57]. The degree of increase in rCBF exceeds that of an increase in the global cerebral metabolic rate of O_2_ [58], which results in a decrease in HHb. Thus, increases in Hbtot (volume) and HbO_2,_ with a decrease in HHb, are expected in activated areas in NIRS measurement. Furthermore, Hoshi *et al.* [59] examined direct effects of changes in CBF on cerebral NIRS signals with an animal model at rest and demonstrated that HbO_2_ was the most sensitive indicator of changes in CBF (*i.e.*, HbO_2_ was augmented by an increase in CBF). Cerebral tissue oxygenation (reflecting the dynamic balance between oxygen supply and oxygen consumption) was recently shown to present a quadratic response to incremental exercise in healthy subjects; it increases from low-to-hard intensities, followed by a plateau or decline toward baseline during severe cycling [43,60,61,62] and running [63] exercises. These previous studies [43,60,61,62,63] provide an overview of the cortical oxygenation changes relative to well-known reliable biomarkers for endurance exercise (lactate or respiratory threshold). During incremental dynamic exercise cerebral oxygenation displayed a non-linear behavior and appears useful for determining an appropriate level of exercise intensity for each individual. In their review, Rooks *et al.* [60] specified that in aerobically trained people, the plateau or a slight decline in cerebral Δ[HbO_2_] concurrent with increasing Δ[HHb] was observed at exhaustive, maximal intensities. Taken together, these findings give the first exercise prescription targeting brain oxygenation improvement according to the trained status of the individuals.

#### 3.1.2. Brain Regions Associated with Muscle Fatigue

Does such a reduction in the NIRS-based cerebral oxygenation during high intensity exercise have a consequence for motor function? There is evidence to support that reduced cerebral oxygenation affects the ability to perform maximal exercise. Motor neuron activity is dramatically influenced by O_2_ availability [64], which plays a pivotal role in the protective mechanisms against muscle fatigue. With exposure to acute hypoxia, CBF and CBF velocity increase to maintain O_2_ delivery. Finally, it has been suggested that cerebral O_2_ supply is well protected in acute hypoxia [65]. However several investigators [66,67] have suggested that a critical reduction in cerebral oxygenation may pose a central limitation to exercise performance by inhibiting cortical activation of efferent motor neurons. In addition, fMRI studies [68,69] reported during fatiguing submaximal constant-load handgrip contractions a progressive increase in the BOLD signal before reaching a plateau toward the end of the task. This plateau, which is consistent with the NIRS reduction in cerebral oxygenation near maximal exercise reported earlier [43,51], can be interpreted as an altered central motor command, *i.e.*, the so-called “central fatigue”. Such an interpretation should take into account that modified BOLD signals can reflect changes in neuronal input and/or processing, and changes in inhibitory and/or excitatory inputs. Techniques allowing regional assessment of cerebral perfusion and oxygenation (such as fMRI and multi-channel NIRS) will be helpful in better describing the topographic localization of cerebral perturbations associated with isolated and whole-body exercises. Recent fMRI studies of the human brain during voluntary muscle fatigue at submaximal intensities levels have shown increased brain activity in areas such as the primary sensory cortex (S1); primary motor cortex (M1); premotor and supplementary motor area (PM, SMA); and prefrontal cortex (PFC) [68,69,70,71]. Altogether, these findings highlight an increased voluntary drive from the cortical centers to spinal alpha motor neurons controlling the working muscles to maintain the target force as fatigue develops. Recently, directionality analysis of fMRI signals originating from different parts of brain during simple *vs.* complex motor tasks has gained a lot of interest in order to reveal the different dynamics of neural network [72]. With diminished force generating the capability of the muscle, functional connectivity between the contralateral primary motor cortex and a number of other cortical regions in the motor control network was shown to increase significantly [73].

As for whole-body exercises, it is evident that NIRS can reveal important information pertaining to the cerebral oxygenation and hemodynamics during static and dynamic resistance exercise. Currently, there is limited research using NIRS examining alterations in cerebral oxygenation and blood volume during sustained lower-body isometric contractions of a muscle group [74,75]. Rupp *et al.* [74] indicated that fatigue during submaximal isometric exercise (ankle extension performed at 40% of the maximal voluntary contraction) is not limited by neuronal activation, but is most likely mediated peripherally in the activated muscle. Likewise, Pereira *et al.* [75] found that the ability to sustain the maximal isometric knee extension (unilateral 1 RM contraction lasting approximately 20 s) was not limited by central neuronal activation: none of the investigated subjects demonstrated a leveling off or decline in cerebral oxygenation during the contraction. The fact that CBV also demonstrated a concomitant increase in almost all subjects supports this finding. In order to elucidate the role of central and peripheral oxygenation status in limiting resistance exercise, the same group of researchers has examined the simultaneous changes in cerebral and muscle hemodynamic using NIRS during two modes of resistance exercise (static and dynamic) at various intensities in men and women [76] and in hypoxia [77]. Their findings emphasized that limitation of knee exercise performance was mediated peripherally in hypoxia and normoxia and was independent of the type and intensity of muscle contraction and gender.

It is noteworthy that in patients who present with restricted CBF, such as those suffering from cerebral ischemia [78,79] an increase of HHb with possible decreases of HbO_2_ during cerebral neural activation has been shown. Interestingly, restriction of (the normal) exercise-induced increase in CBF was shown to alter cerebral neural activation/metabolism [80]. Thus, a decrease in CBF response to neuronal activation can give rise to oxygen deficiency during activation. Loss of vasodilatory response to neuronal activation may be a major contributing factor in some patients.

### 3.2. Chronic Adaptations and Training Effect

Increased CBF to various regions of the brain as a result of exercise may improve neuronal activity [81]. Indeed, exercise promotes angiogenesis (*i.e.*, the growth of new blood vessels linked to neurogenesis) and vascular function in several regions of the brain including the motor cortex [82]. In addition, increased exercise capacity is associated with higher overall levels of neurotrophins and growth factors. In resting conditions, variation in exercise capacity in different rat strains was shown to be associated with variation in BDNF levels [83]. Moreover, rats from strains that voluntarily run long distances had significantly higher levels of BDNF than rats from strains that do not run as far [83]. What about humans?

Repetitive exercise should increase the number of new neurons produced in the human hippocampus—as it greatly increases the number of neurons produced in laboratory animals—but this has not been clearly established. One study examined CBV, a neural correlate of neurogenesis, in mice and humans [84]. They began the study by demonstrating a relationship between dentate gyrus CBV and neurogenesis in mice, as well as an increase in both measures after exercise. When neurogenesis was impaired, via irradiation of the dentate gyrus, exercise no longer increased neurogenesis; nor did it increase dentate gyrus CBV. Based on these data, the authors suggested that exercise-induced increases in dentate gyrus CBV can be considered a neural correlate of exercise-induced neurogenesis in humans. The researchers then studied these relationships in healthy sedentary humans. The subjects were engaged in twelve weeks of physical training, with four one-hour sessions of aerobic exercise per week. As expected, this exercise program greatly increased CBV in the dentate gyrus, where the new hippocampal neurons are generated. Furthermore, each individual’s change in dentate gyrus CBV correlated with the change in their maximal VO_2_ after physical training. Emerging portable and miniaturized fNIRS systems open new perspectives to studying CBV and motor function in realistic environments during exercise training. Moreover, future fMRI and fNIRS studies have the possibility to delineate potential brain response signatures to study the response of individuals to demanding physical conditions in studies of fitness training effects on motor performance.

Cerebral metabolism and CBF increase during exercise [10,53], signifying the increased metabolic demand on neuronal cells during training and the likely underlying mechanism through which neuroprotection is obtained. The angiogenic changes that occur following exercise preconditioning provide the brain with an enriched vascular bed with an enhanced ability for proper CBF and glucose delivery to neurons, yielding more tolerance to reperfusion injury in the setting of ischemia/reperfusion. Exercise training transforms the neurovascular system of the neurovascular unit, developing a vital metabolic response network in response to ischemia. Under normal conditions, angiogenesis and endothelial cell proliferation are negligible in the adult brain [85]. Previous studies have shown that physical activity on a treadmill increases blood vessel density in the brain [86,87], and forced exercise on a treadmill induces cortical and striatal angiogenesis [88]. In addition to these increases in angiogenesis, exercise preconditioning also increases arteriogenesis, which promotes CBF. These changes not only facilitate increased glucose and oxygen delivery, but they have also been shown to reduce brain damage, as well [88]. Several regulator proteins, namely vascular endothelial growth factor (VEGF) and angiopoietins (Ang) 1 and 2 are expressed in greater abundance following exercise training, and these changes lead to increased blood vessel density [88]. In addition to correlating with increased CBF and glucose utilization, the angiogenesis and arteriogenesis observed after exercise preconditioning are, indeed, associated with decreased neuronal cell death following ischemia/reperfusion injury [89]. 

While it is now well accepted that regular physical exercise can be used as an efficient treatment strategy to improve CBF [90,91], some issues have still to be resolved. No specific training guidelines have been established through human studies. Larson *et al.* [92] revealed that moderate intensity and duration of exercise, as opposed to mild or strenuous, correlate with better outcomes and life improvements. These preliminary findings show that moderate exercise over a longer time period conveys more efficient and greater neuroprotection than more vigorous exercise over shorter time periods.

## 4. Associations between Brain Activity Changes and Motor Function Recovery

Determination of brain activation patterns associated with functional force production and modulation has important implications in clinical rehabilitation. Damage to motor areas of the brain, caused by stroke, can produce devastating motor deficits. After stroke, reorganization of the brain’s motor system has been identified as one of the fundamental mechanisms involved in recovery of motor control. Ischemia has profound effects on CBF levels due to impaired pressure autoregulation. A loss of CBF regulatory capacity can be attributed either to damage of the control system (cerebral vessels) or of the feedback mechanisms involved in the brain’s hemodynamic control. At this time, CBF becomes pressure-dependent and, therefore, small changes in mean arterial pressure can have profound effects on CBF and CBV. Thus, the neurovascular control of CBF is disrupted during ischemia, with the loss of coupling between neural activity and hemodynamic effects [93]. Among neuroprotective strategies that have multiple mechanisms of action including augmenting CBF, one attractive non-pharmacological and inexpensive option for ischemic stroke is exercise training.

### 4.1. Reorganization of Brain Activation after Brain Injury

Indeed, after a brain injury, such as stroke, one mechanism that the CNS uses to compensate for its loss is through neural plasticity. For example, after cortical injury, the greater the damage that occurs to the M1, the greater the reorganization of intact areas, like the premotor cortex [94]. Ischemic brain injury is a consequence of a severe reduction in blood supply to the affected region. The resultant low tissue oxygen tension after ischemia often leads to compensatory neovascularization or angiogenesis, in order to meet the metabolic demand [95]. Functional reorganization of the CNS is thought to be one of the fundamental mechanisms involved in recovery after neurological injury. Past animal studies have established that structural plasticity can occur in the damaged cortex and connected brain areas, and that functional recovery is associated with this plasticity [96]. Similarly, neuroimaging studies in humans have demonstrated that reorganization of brain activation relates to functional outcome after stroke [97]. Stroke patients with a higher density of blood vessels appear to have reduced morbidity and survive longer [98]. Interestingly, functional imaging of stroke patients shows increased CBF and metabolism in tissue surrounding focal brain infarcts [99]. These data suggest that restoration of cerebral microvascular circulation is important for functional recovery after a stroke. Aerobic exercise can increase capillary density, and, in turn, it may spare neuronal function by maintaining adequate glucose and oxygen availability to neurons. In rats, exercise has been reported to increase capillary density in motor areas of the brain. For instance, one month of exercise (voluntary or forced) increased capillary density in the cerebellar molecular layer [100], in the motor cortex, as well as capillary perfusion [86,87]. While past studies demonstrated clearly that reorganization of brain activation occurs during movement after stroke, many employed tasks are not performed against resistance, such as finger tapping [99]. Unfortunately, these tasks may not be as useful as tasks involving active movement against resistance, as these latter tasks can allow different forces to be produced and thus are more highly relevant to activities of daily living (e.g., lifting a book, holding a cup). 

### 4.2. Exercise-Induced Neuroprotection

While various modalities, such as treadmill running, voluntary running, or simple and complex exercise, have been shown to provide variable amounts of neuroprotection, further training components need to be proposed and tested. When comparing forced exercise on a treadmill to voluntary running on a running wheel, rats forced to exercise on a treadmill tend to have better neurologic outcomes after stroke [101]. Forced exercise can be defined as a mode of aerobic exercise in which exercise rate is augmented mechanically to assist the subject in achieving and maintaining an exercise rate that is greater than their preferred voluntary rate of exercise. Recent data indicate that forced exercise has neuroprotective properties [102] and improves motor function [103] in a rodent model of Parkinson’s disease. In humans, there is a paucity of studies proposing such a training mode. This neuroprotection in forced exercise subjects induces lessened neurologic deficit, increased neurogenesis, and cerebral metabolism [102,104]. For patients wanting to derive motor benefits from exercise, assistance is required to achieve a rate of exercise that, for instance, triggers the release of neurotrophic factors. Forced exercise appears to have neuroprotective properties and to improve motor function in mice. The complete and most convincing study in humans comes from Alberts *et al.* [105], where the authors developed tandem cycling as a form of forced exercise in humans and used it in an eight-week exercise intervention (three 1-h exercise sessions per week, 40 min main exercise, intensities corresponding of 60%–80% of individualized target heart rate) for improving Parkinson’s disease motor function. In this study, enhanced fine motor function of the upper extremities after lower extremity exercise supported the hypothesis that forced exercise alters central motor processing as compared to voluntary exercise. Exercise in Parkinson’s patients led to an increase in cortical and subcortical activation [105]. In short, animal and human studies suggest that exercising at a high rate induces better improvements in motor functions.

In addition, differences have also been established when comparing simple (repetitive movements) and complex (enriched activities which required both balance and coordination) exercise. Following ischemia/reperfusion injury, rats preconditioned with complex exercise demonstrated increased synaptogenesis and improved neurologic outcomes when compared to simple treadmill exercise [106]. Simple exercise training also alleviates much of the injury following ischemia/reperfusion, but its benefit is less pronounced than what is observed with complex exercise training. Although no human studies have definitively shown the most effective means of exercise for enhanced neuroprotection, these animal studies provide a solid framework for the development of appropriate exercise regimens to set in humans. Moderate exercise intensity, including components of balance, coordination and stress, taking place over a sustained duration, seems promising for being the most neuroprotective when compared to other exercise modalities and intensities. One main issue to solve is to track the ongoing neuroplasticity during the time course of exercise training. Brain monitoring at home, in remote locations or during tasks that require substantial motion (e.g., gait training), is generally precluded.

### 4.3. NIRS for Monitoring Training Programs

Among available neuroimaging methods (as previously described), NIRS-based techniques promise to enable a wider scope of clinical and research studies. Several investigators have used NIRS to evaluate cerebral oxygenation and blood volume changes in patients with neurological disorders, including stroke [45,107]. By using NIRS-derived parameters (increase in CBV and CBF) to monitor task effect, different patterns with regards to the rehabilitation tasks used suggest diverse efficacy for facilitating cerebral activation. The first cross-sectional study using NIRS in a rehabilitation setting was carried out by Saitou *et al.* [107], who investigated 44 stroke patients and 24 control subjects. In their study, the authors proposed 13 rehabilitation tasks, including cycle ergometer, passive wrist and finger extension, passive movement of affected upper limbs, free gait, reciprocal extension of knee joints, *etc.* Interestingly, the authors found a significant percentage of subjects exhibiting major changes in CBV over the cortex. Usually, one of the rehabilitation programs to improve the neuromuscular control of ambulation in stroke patients is the leg cycling exercise. Passive cycling motion (without active muscle contractions) also appears as an alternative program for patients with severe motor dysfunction. Recently, however, Lin *et al.* [45] investigated whether changes in cortical activation patterns accompany improvements in cycling performance in stroke patients. The authors discovered that both active and passive cycling motions activated similar cortical regions, such as the primary sensory and motor cortices. Facilitating motor recovery and brain neuroplasticity in stroke patients with intensive exposure to somatosensory stimulation induced by passive cycling training is thus warranted. 

## 5. Conclusion

Over the past years, a number of studies have emerged that have attempted to investigate the effects of exercise on brain function after injury or otherwise. While exercise guidelines for adults have traditionally focused on achieving musculoskeletal and cardiopulmonary benefits with training, more recent attention has shifted to exercise as a means of maintaining or increasing brain health. Although the methodologies of these studies were different, they globally showed that increased task-related activation in the brain occurs and is accompanied by an improved motor function. Among the available functional imaging, NIRS-related parameters can be useful markers of exercise change during one bout or a training program. The angiogenic changes that occur following exercise preconditioning provide the brain with an enriched vascular bed with an enhanced ability for proper CBF and glucose delivery to neurons while exercise training is able to transform the neurovascular system of the neurovascular unit. Based on their neural responses, forced exercise, rhythmic exercise, and complex motor tasks appear to be interesting motor training protocols.
